# Supplementary figures and images for: DIP2 is a unique regulator of diacylglycerol lipid homeostasis in eukaryotes
Source: eLife. 2022 Jun 29;11:e77665. doi: 10.7554/eLife.77665 (PMC9342972; doi:10.7554/eLife.77665)

Figure 1-figure supplement 2A  
and 2B-source data 1

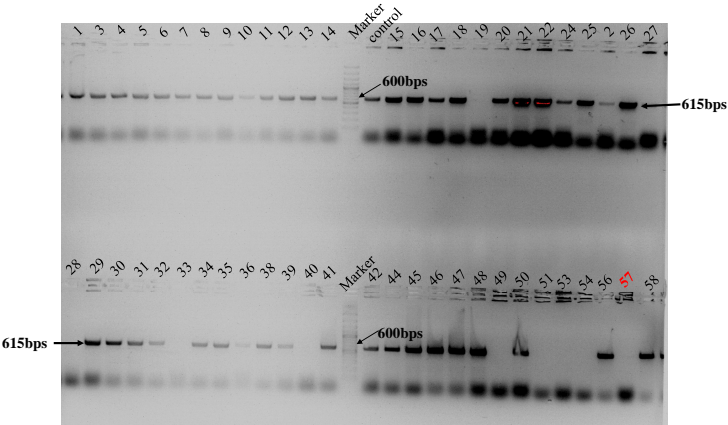

Image-1

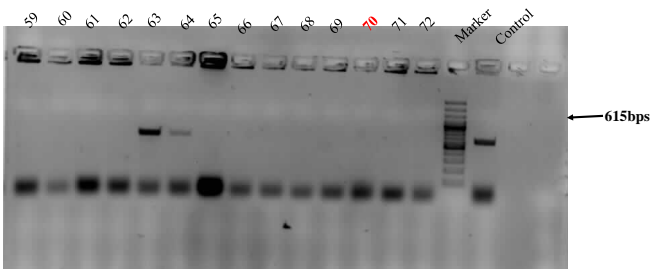

Image-2

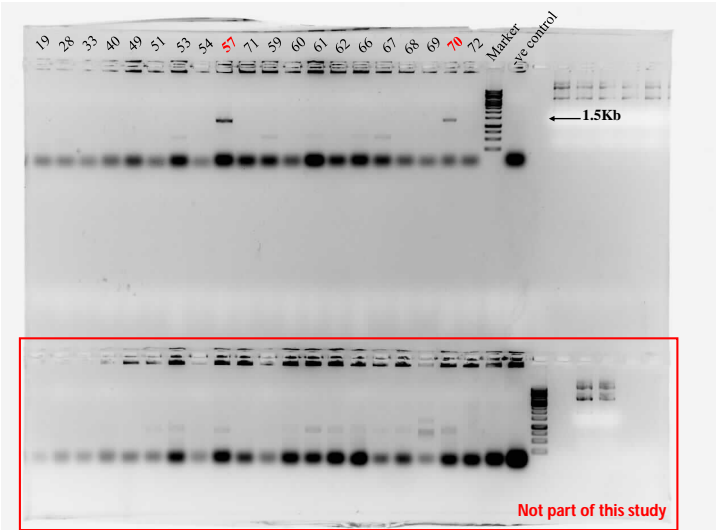

Image-3

Supplement: Figure 1—figure supplement 2—source data 1. [file elife-77665-fig1-figsupp2-data1.pdf]

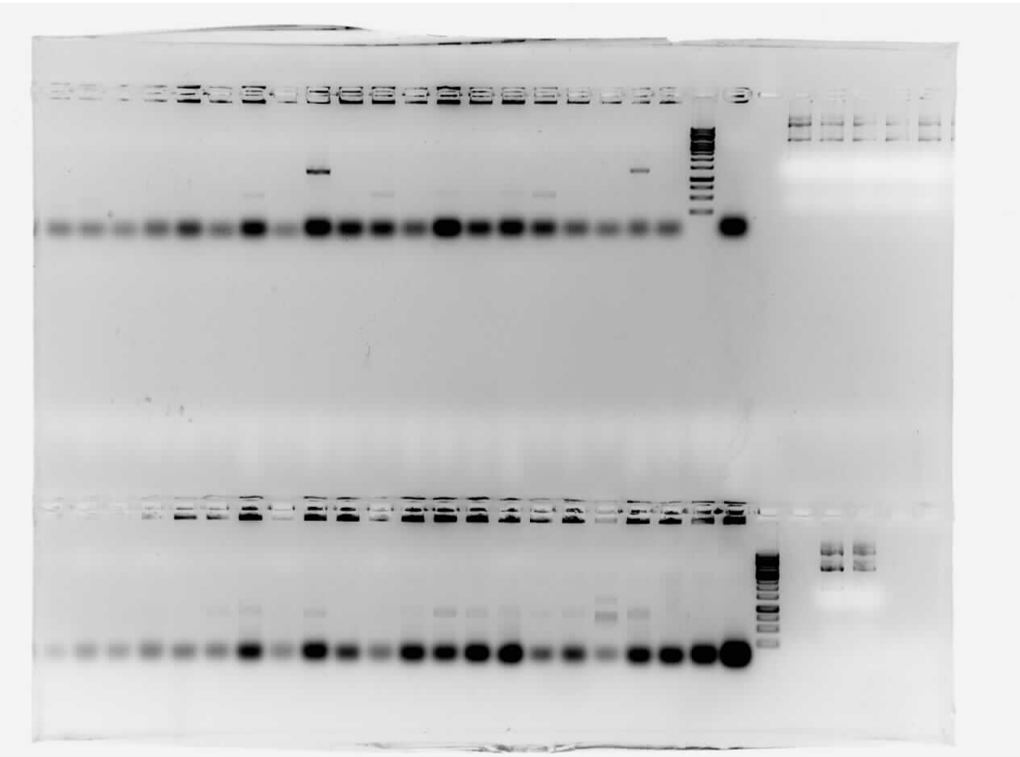

Supplement: Figure 1—figure supplement 2—source data 2. [file elife-77665-fig1-figsupp2-data2.zip › Figure 1-figure supplement 2A and 2B-source data 1 image3.tif]

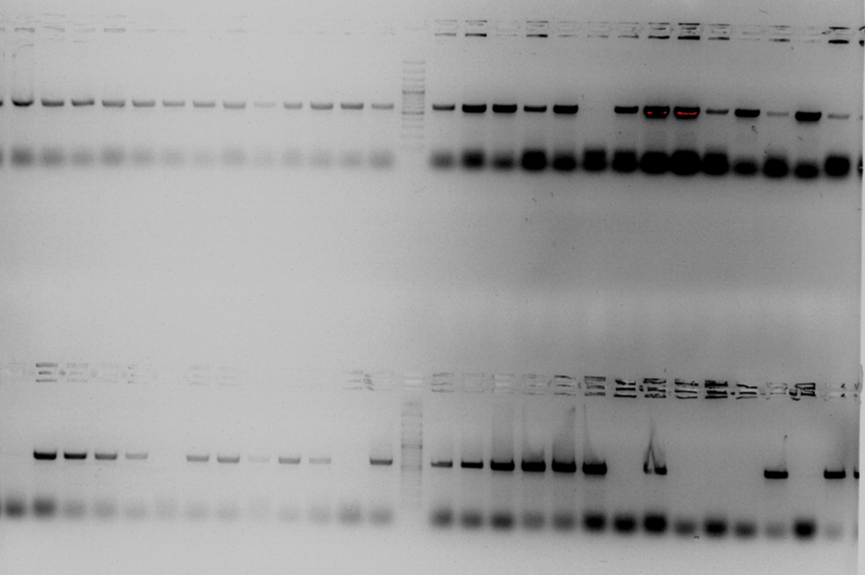

Supplement: Figure 1—figure supplement 2—source data 2. [file elife-77665-fig1-figsupp2-data2.zip › Figure 1-figure supplement 2A and 2B-source data 1 image1.tif]

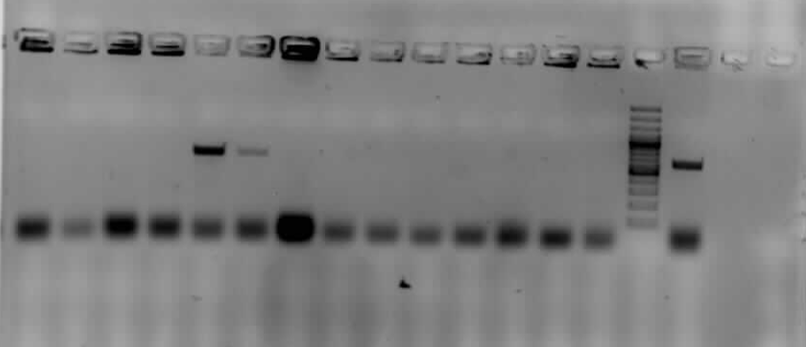

Supplement: Figure 1—figure supplement 2—source data 2. [file elife-77665-fig1-figsupp2-data2.zip › Figure 1-figure supplement 2A and 2B-source data 1 image2.tif]

# Figure 3-figure supplement 2B- source data 1

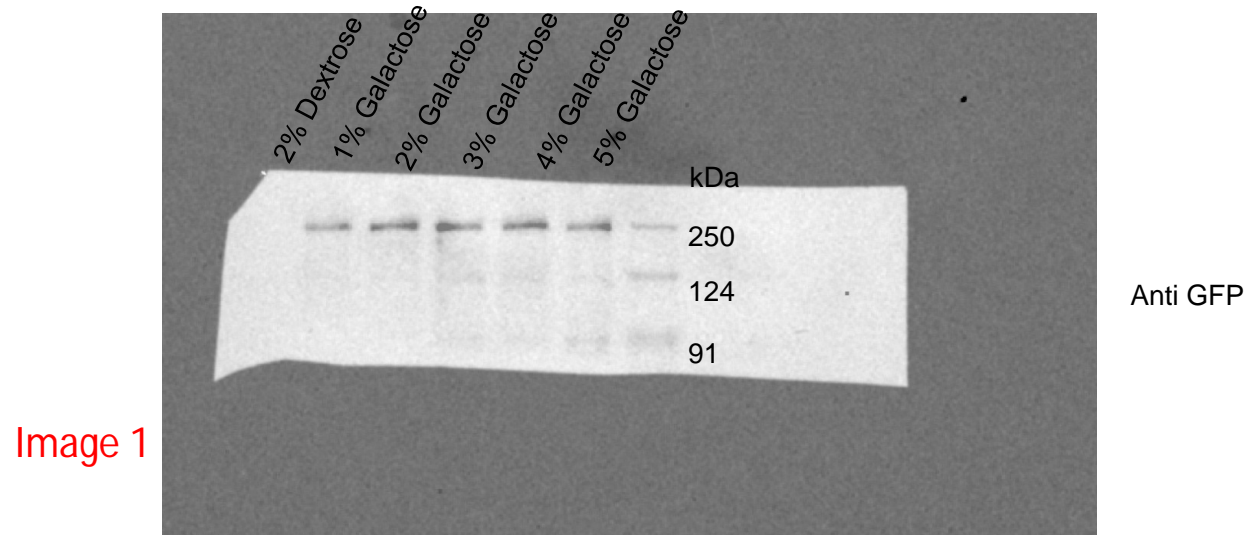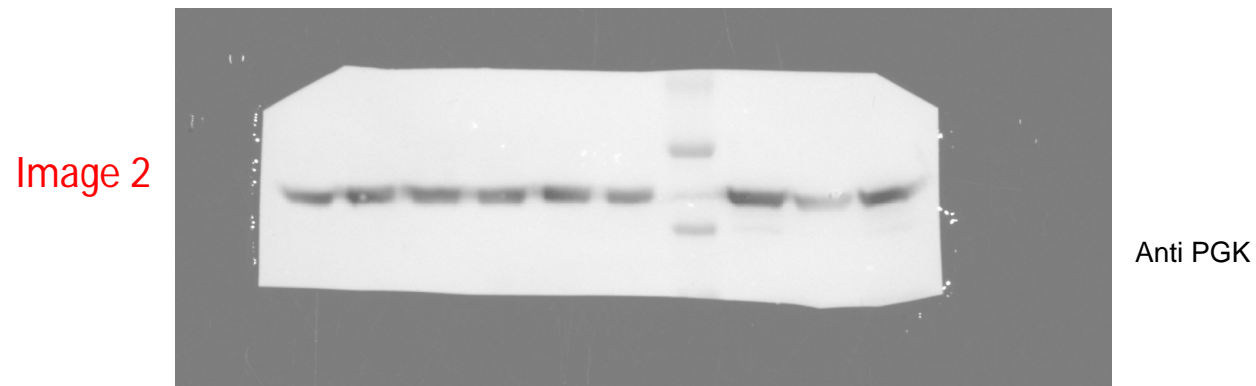

Supplement: Figure 3—figure supplement 2—source data 1. [file elife-77665-fig3-figsupp2-data1.pdf]

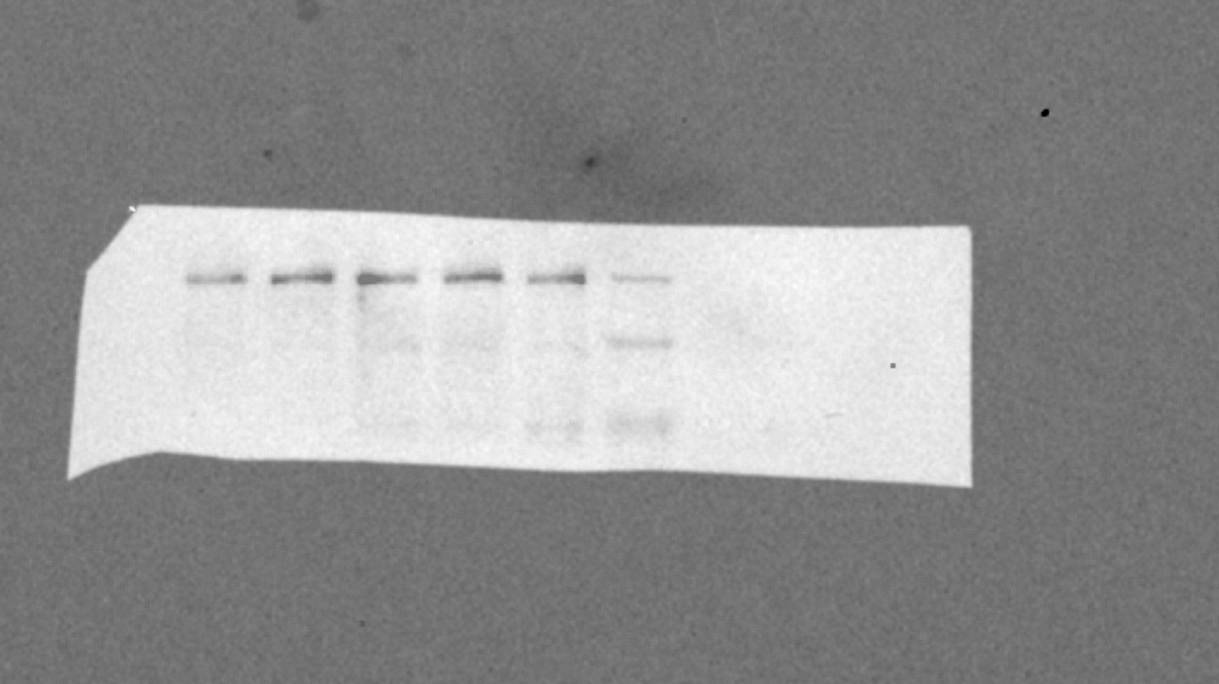

Supplement: Figure 3—figure supplement 2—source data 2. [file elife-77665-fig3-figsupp2-data2.zip › Figure 3-figure supplement 2B- source data 1 image 1.tif]

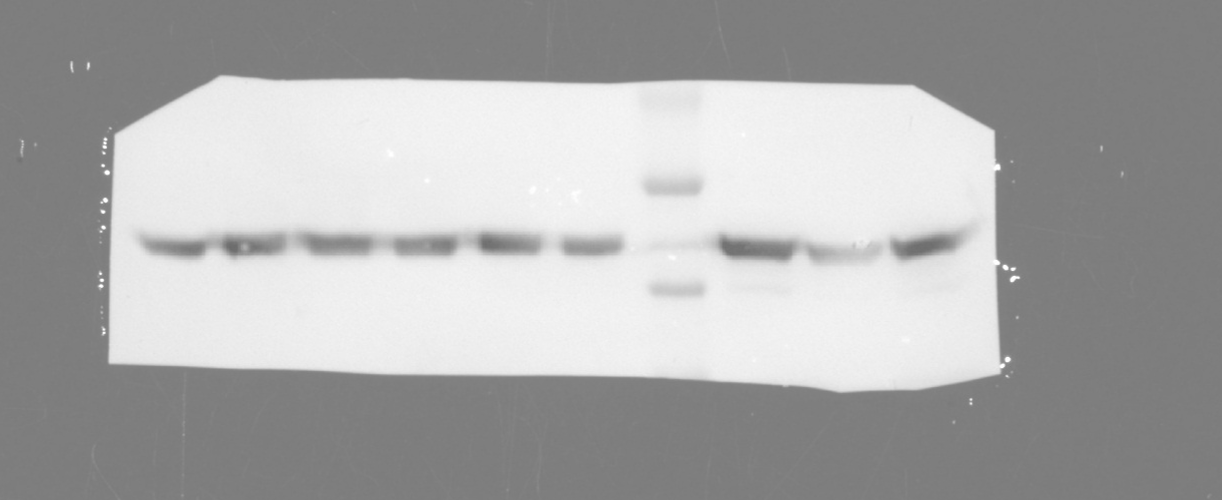

Supplement: Figure 3—figure supplement 2—source data 2. [file elife-77665-fig3-figsupp2-data2.zip › Figure 3-figure supplement 2B- source data 1 image 2.tif]

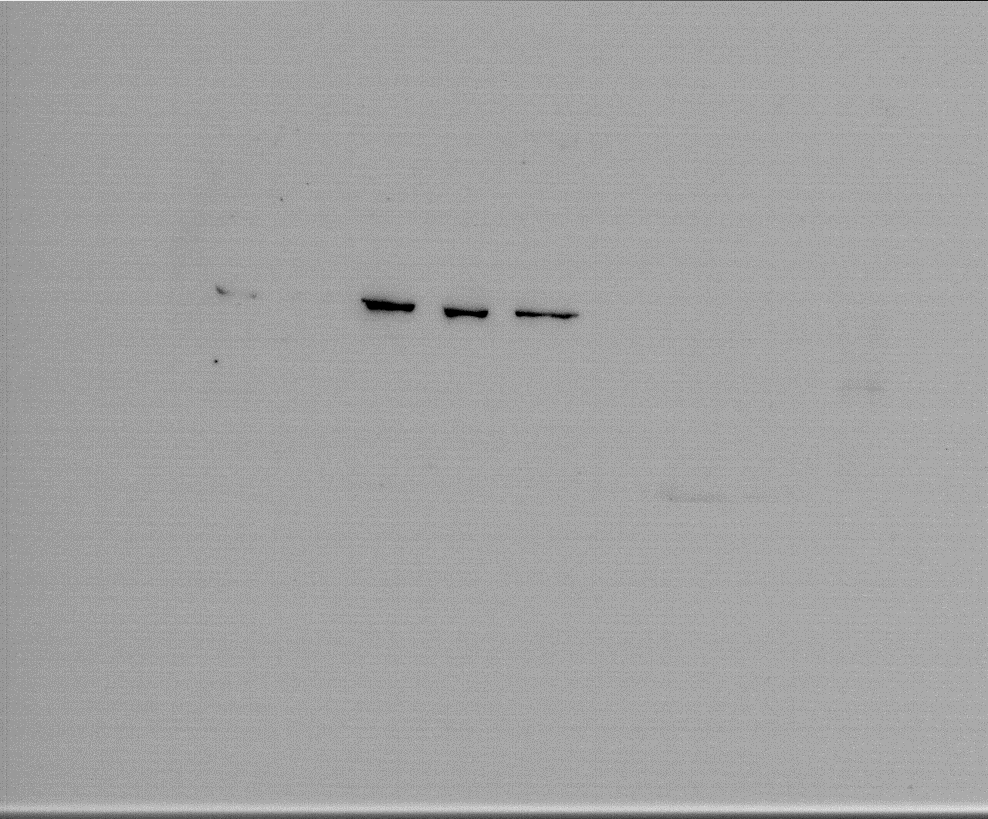

Supplement: Figure 5—figure supplement 1—source data 2. [file elife-77665-fig5-figsupp1-data2.zip › Figure 5-figure supplement 1E -source data 1 image 1.tif]

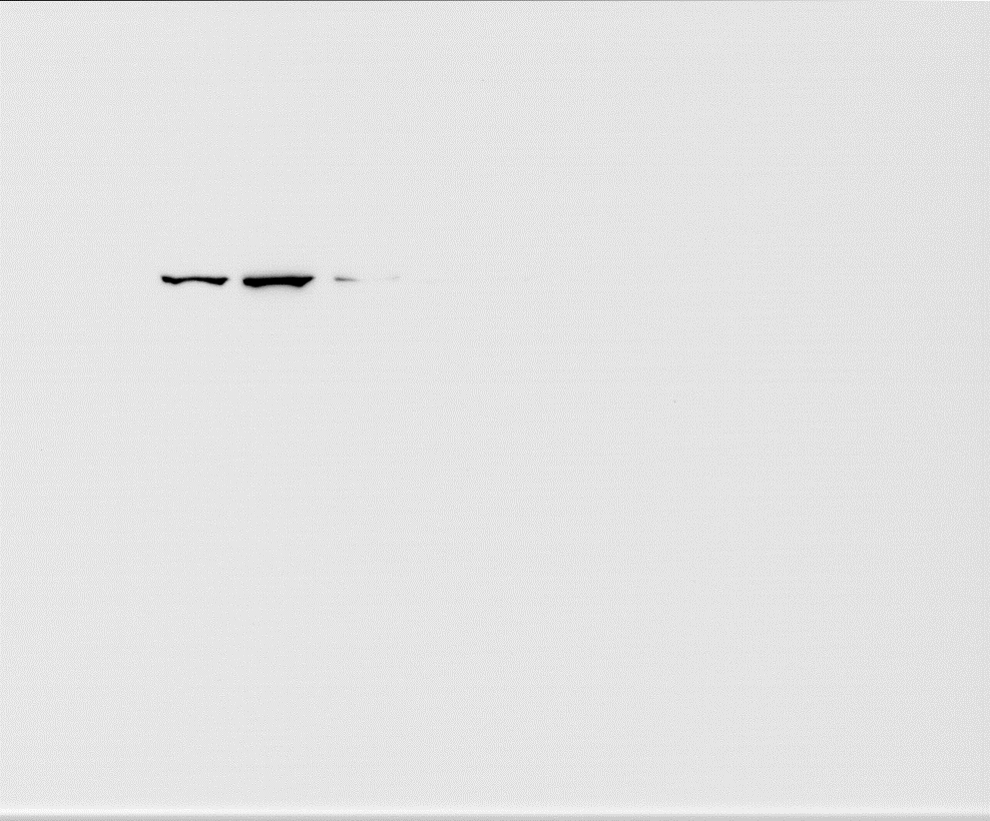

Supplement: Figure 5—figure supplement 1—source data 2. [file elife-77665-fig5-figsupp1-data2.zip › Figure 5-figure supplement 1E -source data 1 image 2.tif]

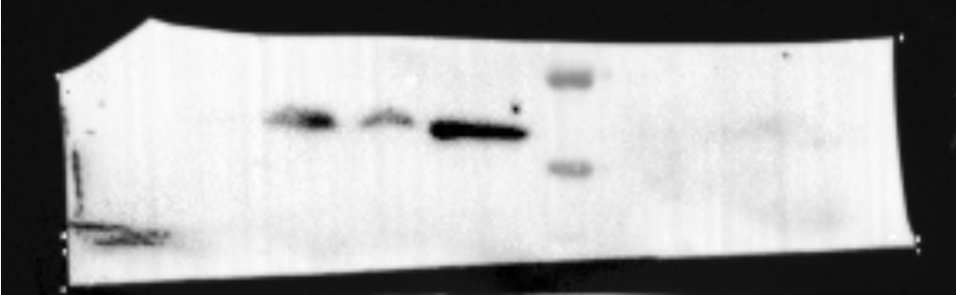

Supplement: Figure 5—figure supplement 1—source data 2. [file elife-77665-fig5-figsupp1-data2.zip › Figure 5-figure supplement 1E -source data 1 image 3.tif]

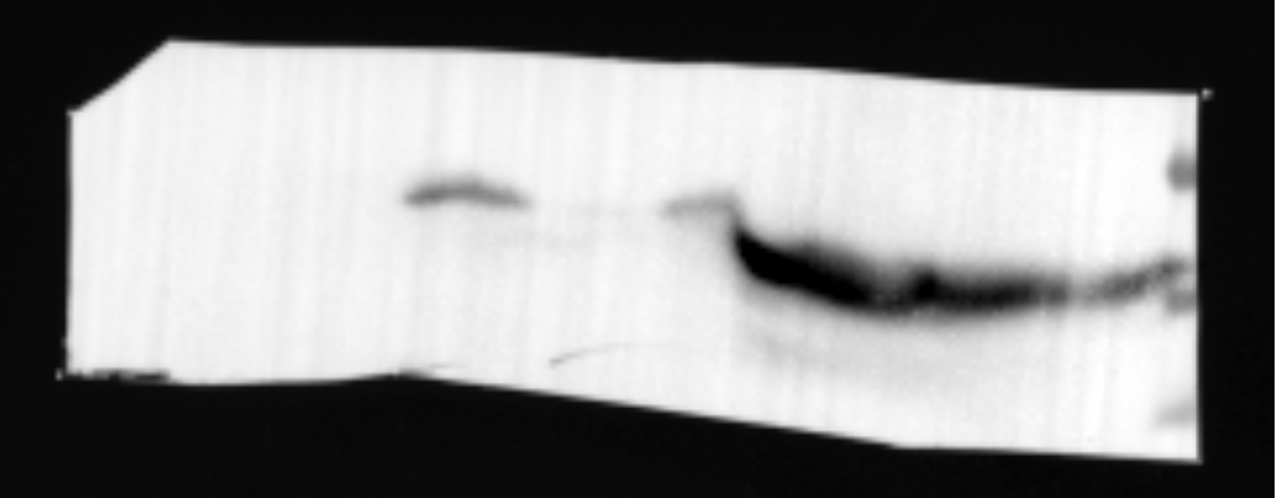

Supplement: Figure 5—figure supplement 1—source data 2. [file elife-77665-fig5-figsupp1-data2.zip › Figure 5-figure supplement 1E -source data 1 image 4.tif]

# Figure 7-figure supplement 1B

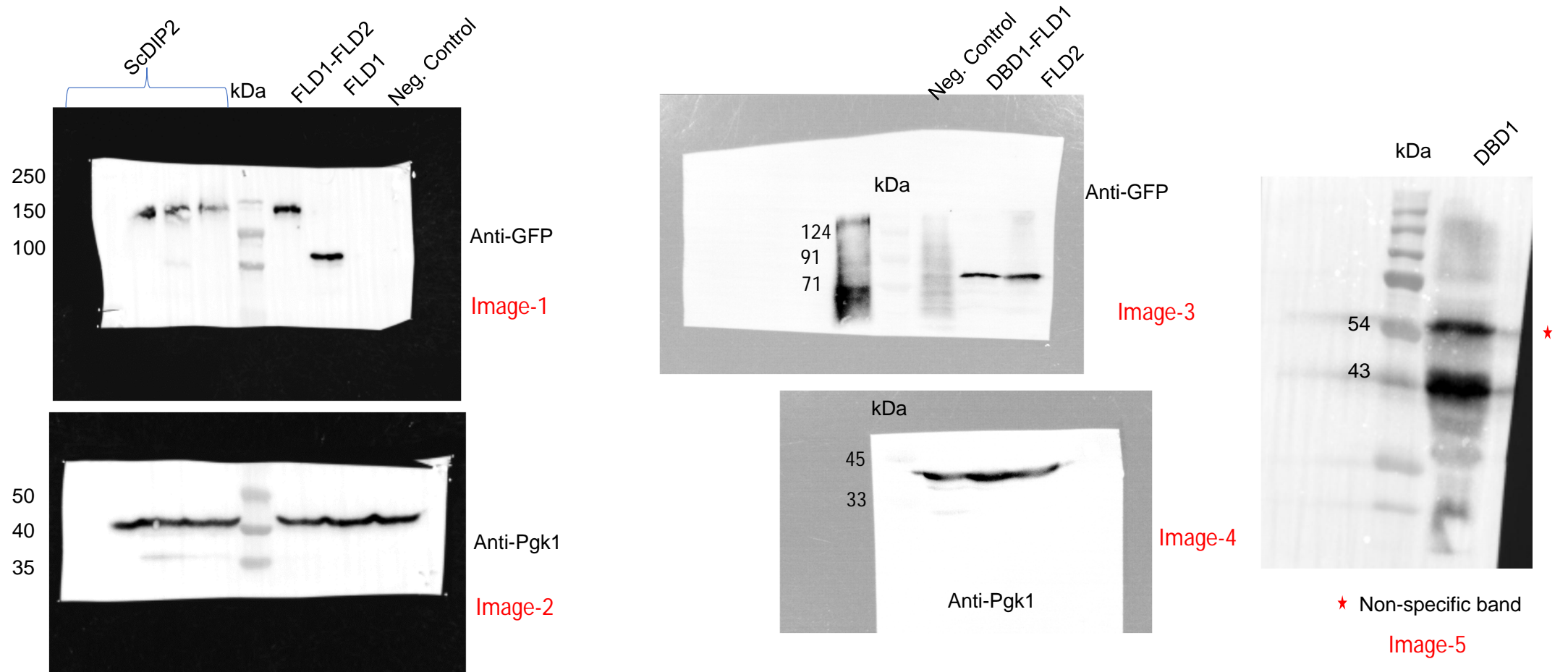

Supplement: Figure 7—figure supplement 1—source data 1. [file elife-77665-fig7-figsupp1-data1.pdf]

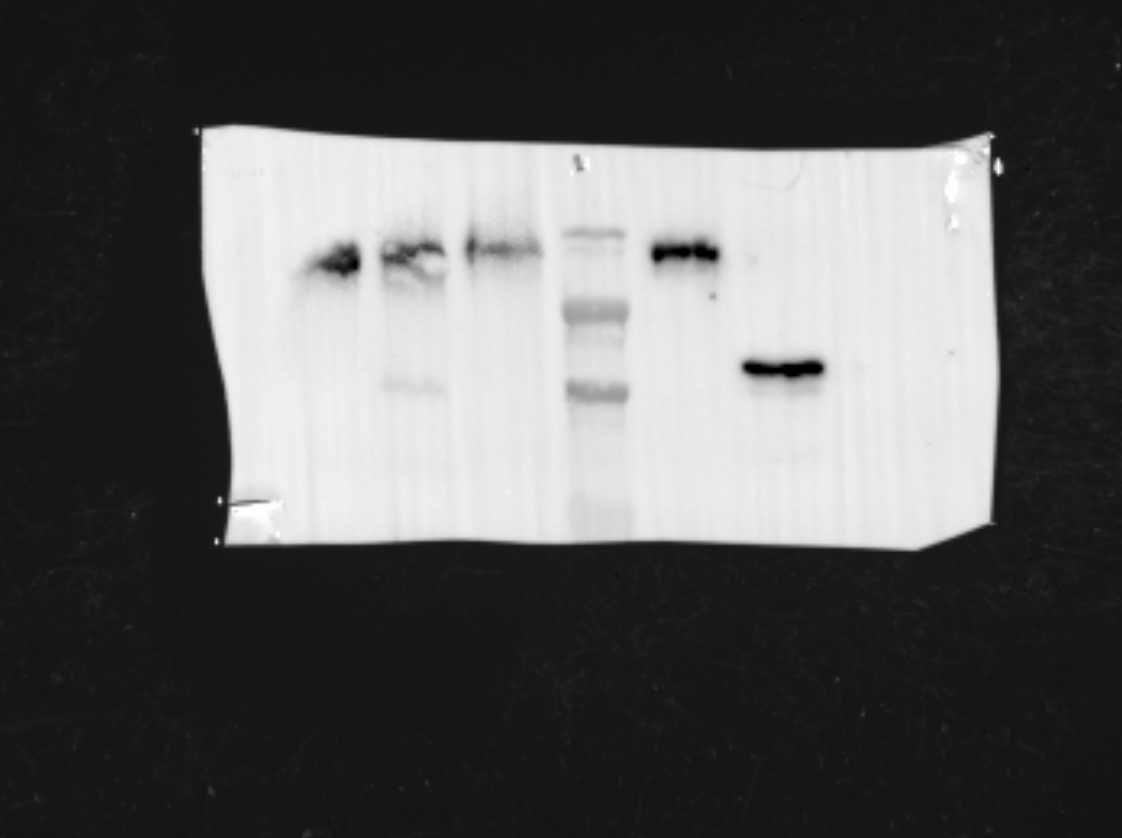

Supplement: Figure 7—figure supplement 1—source data 2. [file elife-77665-fig7-figsupp1-data2.zip › Figure 7-figure supplement 1B-source data 1 image 1.tif]

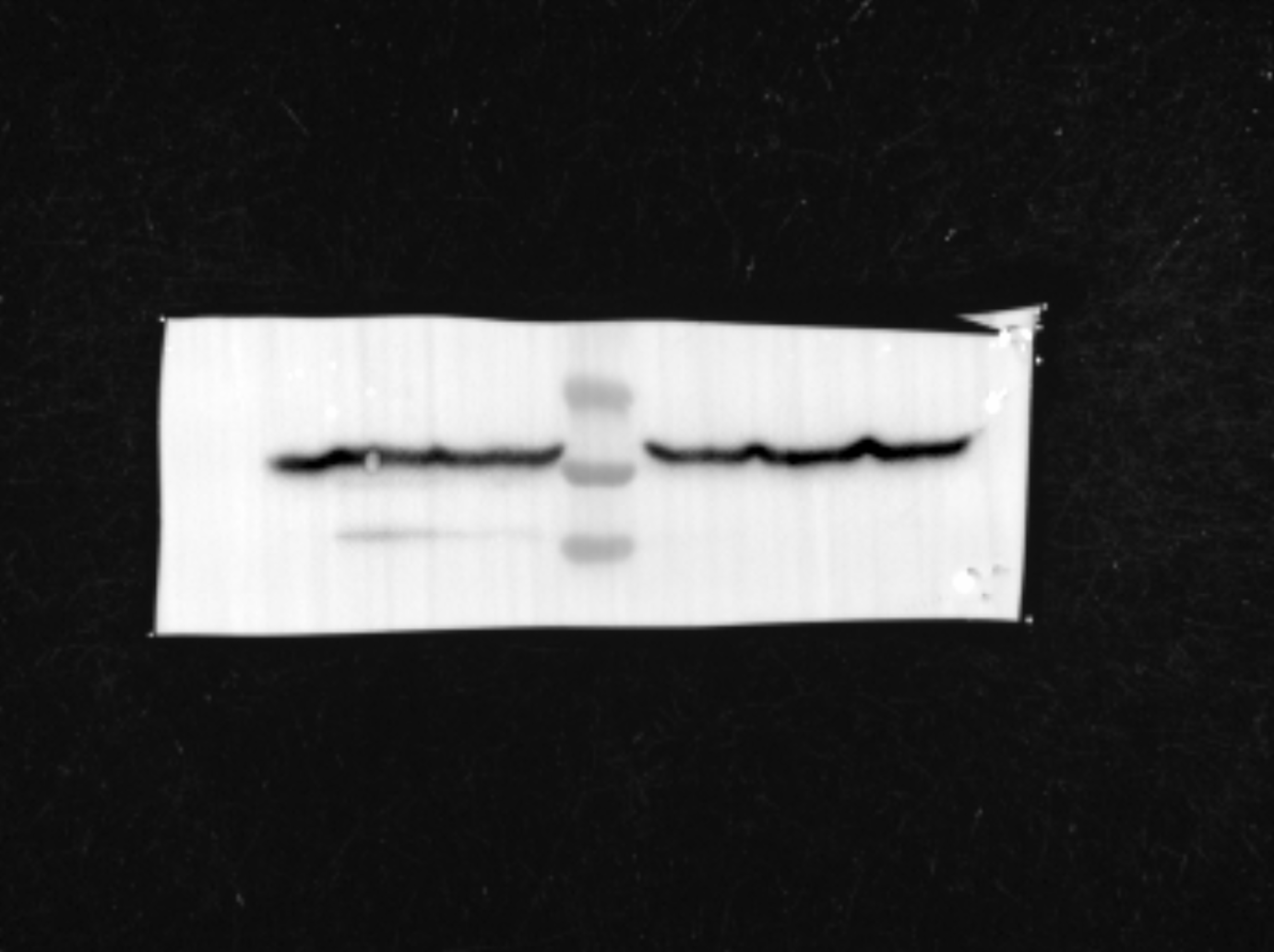

Supplement: Figure 7—figure supplement 1—source data 2. [file elife-77665-fig7-figsupp1-data2.zip › Figure 7-figure supplement 1B-source data 1 image 2.tif]

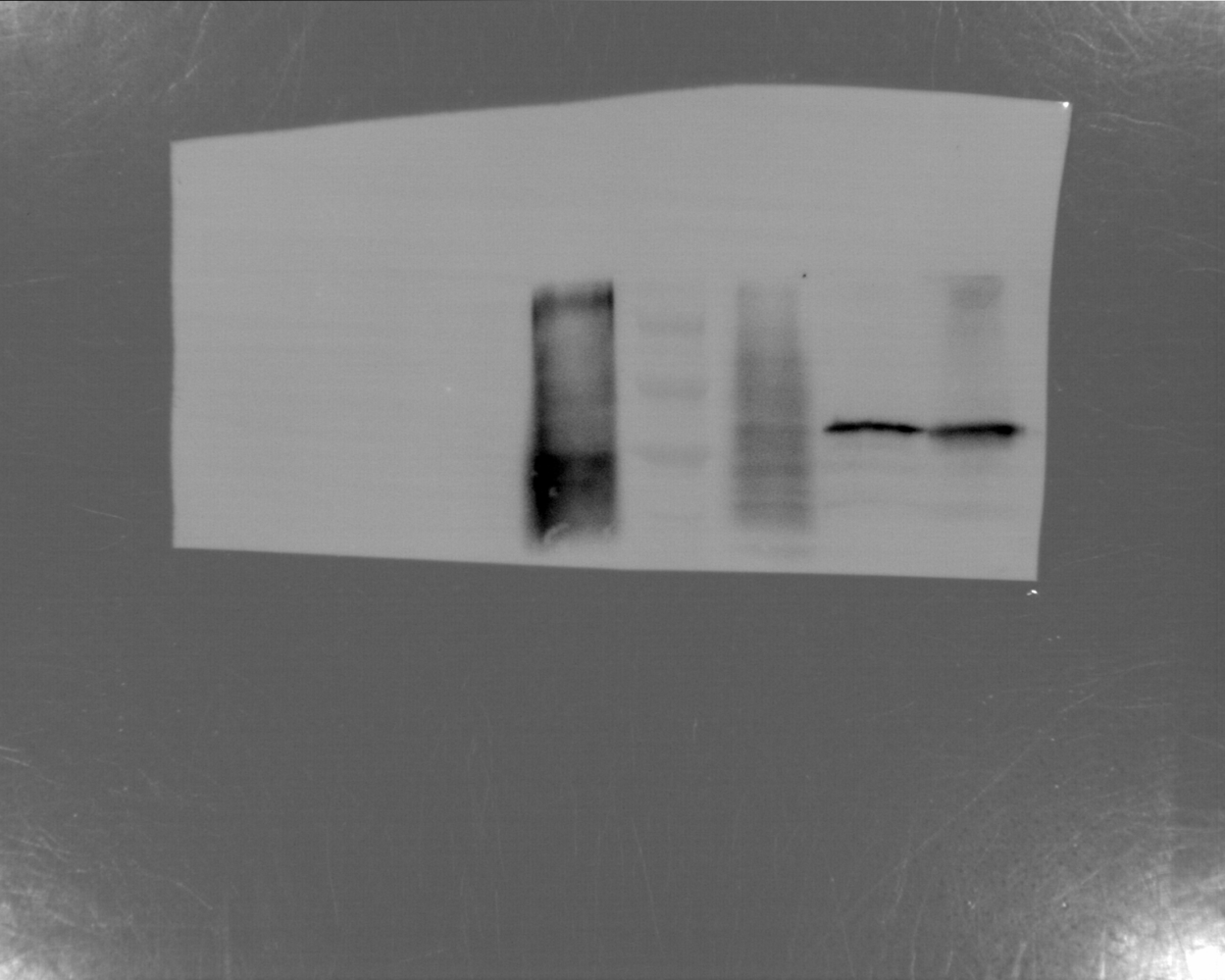

Supplement: Figure 7—figure supplement 1—source data 2. [file elife-77665-fig7-figsupp1-data2.zip › Figure 7-figure supplement 1B-source data 1 image 3.tif]

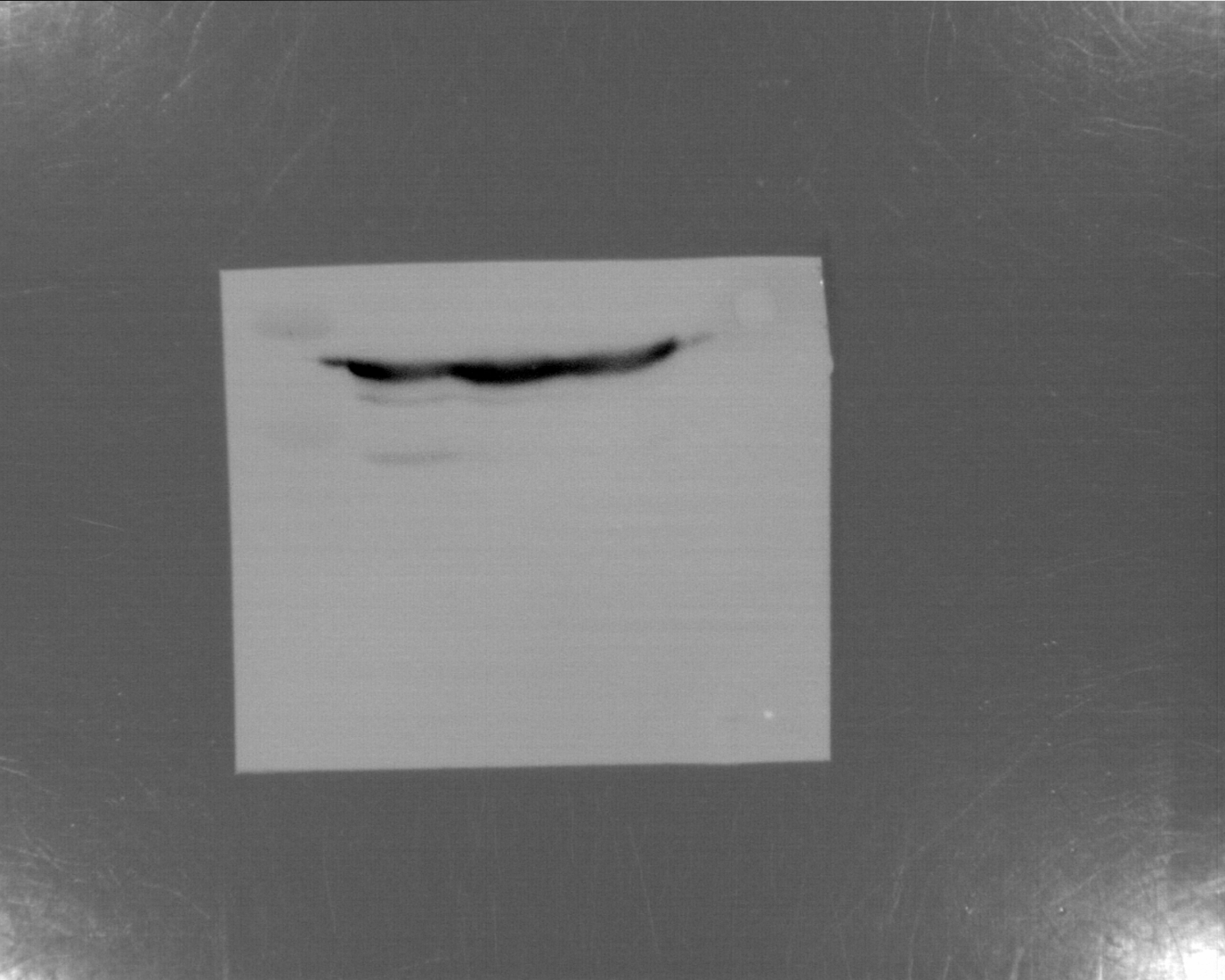

Supplement: Figure 7—figure supplement 1—source data 2. [file elife-77665-fig7-figsupp1-data2.zip › Figure 7-figure supplement 1B-source data 1 image 4.tif]

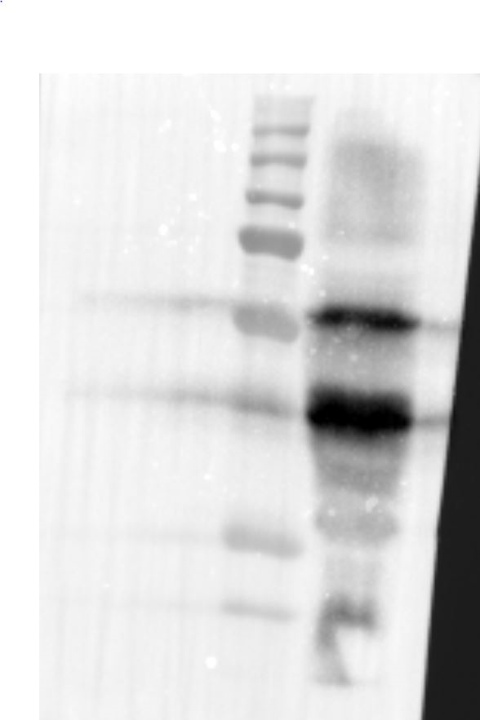

Supplement: Figure 7—figure supplement 1—source data 2. [file elife-77665-fig7-figsupp1-data2.zip › Figure 7-figure supplement 1B-source data 1 image 5.tif]

## Figure 7-figure supplement 2C- source data 1

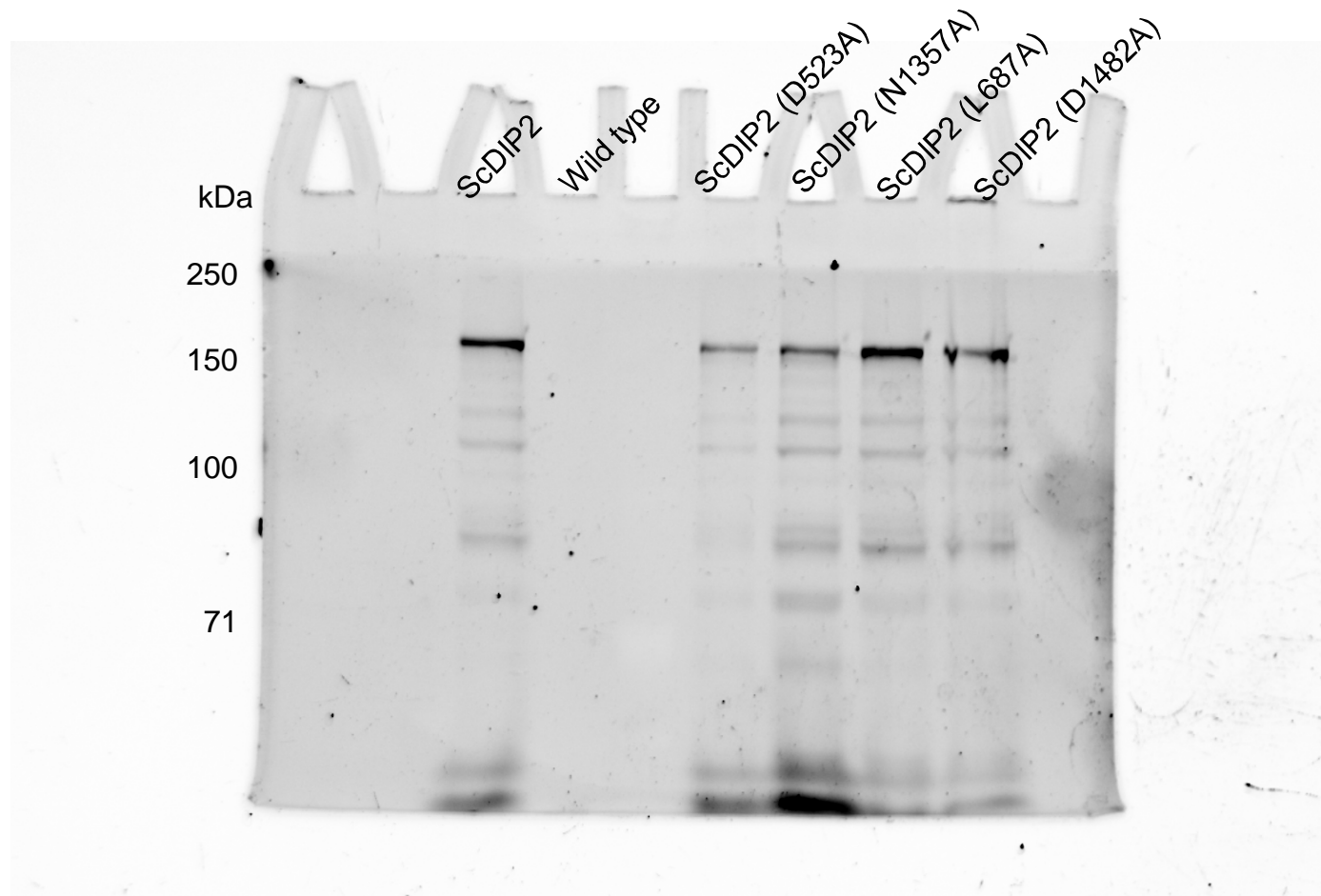

Supplement: Figure 7—figure supplement 2—source data 1. [file elife-77665-fig7-figsupp2-data1.pdf]

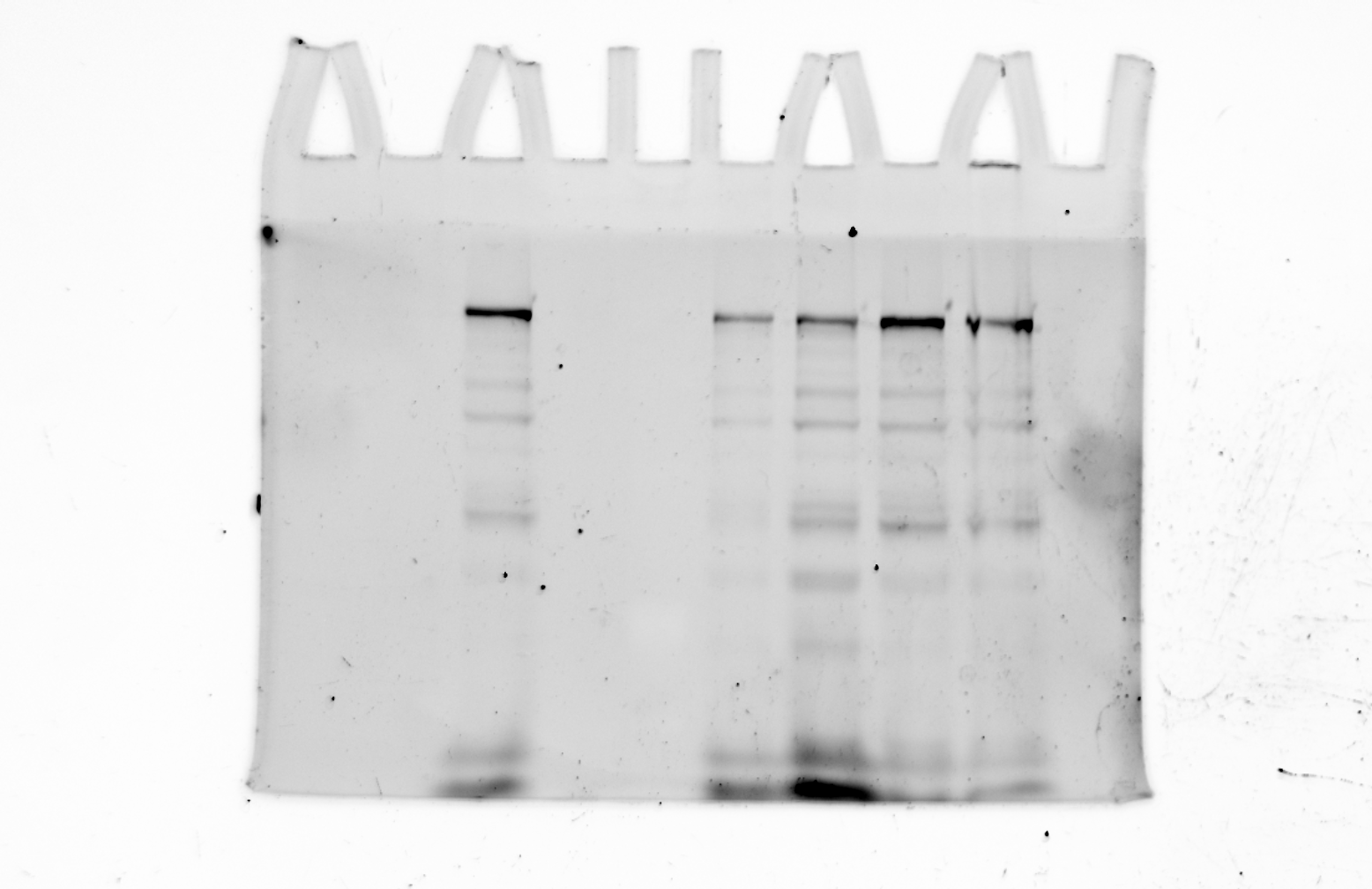

Supplement: Figure 7—figure supplement 2—source data 2. [file elife-77665-fig7-figsupp2-data2.zip › Figure 7-figure supplement 2C- source data 1.tif]
