# Supplementary material for: DIP2 is a unique regulator of diacylglycerol lipid homeostasis in eukaryotes
Source: eLife. 2022 Jun 29;11:e77665. doi: 10.7554/eLife.77665 (PMC9342972; doi:10.7554/eLife.77665)
Supplement: Figure 5—figure supplement 1—source data 1. [file elife-77665-fig5-figsupp1-data1.pdf]

Figure 4-figure supplement 1B -source data 1

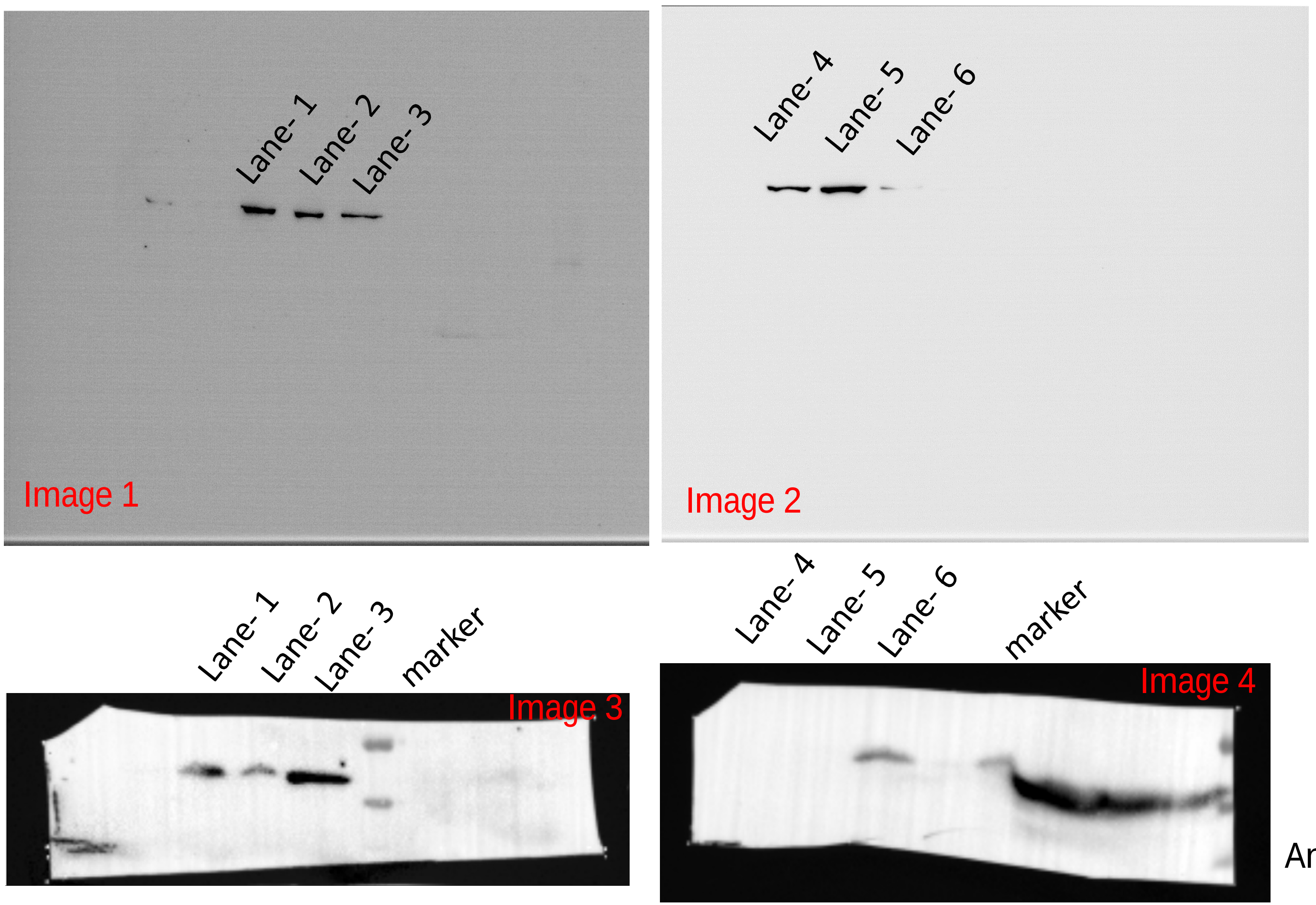

Anti-GFP

Anti-PGK1

- Lane-1: not part of study
- Lane-2: total lysate
- Lane-3: crude organelle lysate (devoid of cell wall debris and nuclei)
- Lane-4: not part of study
- Lane-5: crude membrane fraction
- Lane-6: membrane-less supernatant
