# Supplementary material for: DIP2 is a unique regulator of diacylglycerol lipid homeostasis in eukaryotes
Source: eLife. 2022 Jun 29;11:e77665. doi: 10.7554/eLife.77665 (PMC9342972; doi:10.7554/eLife.77665)
Supplement: Supplementary file 1. [file elife-77665-supp1.docx]

**Supplementary Table 1:**

|  | **Organism** | **Mutation** | **Phenotype** | **Disease/Pathogenesis** | **Reference** |
| --- | --- | --- | --- | --- | --- |
|  |  |  |  |  |  |
| ***Fungi*** | *Cochliobolus heterostrophus* | ΔDIP2 | Reduced or loss of virulence | Southern leaf blight | (Lu et al., 2003) |
|  | *Cochliobolus victoriae* |  | Reduced or loss of virulence | Victoria blight |  |
|  | *Gibberella zeae* |  | Reduced or loss of virulence | Fusarium head blight, |  |
|  | *Magnaporthe oryzae* | ΔDIP2 | Loss of Virulence | Rice blast/blight | (Wang et al., 2016) |
|  | *Coccidioides posadasii* | ΔDIP2 | Loss of Virulence | Valley fever | (Narra et al., 2016; Shubitz et al., 2018) |
|  |  |  |  |  |  |
| ***Animals*** | *Caenorhabditis elegans* | ΔDIP2 | Increased ectopic neurite sprouting, branching and axon regeneration | - | (Noblett et al., 2019) |
|  | *Drosophila melanogaster* | ΔDIP2 | Axon branching and guidance defect in mushroom-body neurons | - | (Nitta et al., 2017) |
|  | *Mus musculus* | ΔDIP2a | Defective dendritic spine morphogenesis and reduced synaptic transmission | Autism-like behaviour | (Ma et al., 2019) |
|  |  |  | Diet dependent growth defects | - | (Kinatukara et al., 2020) |
|  |  | ΔDIP2b | Excessive axonal outgrowth, Reduced synaptic transmission | - | (Xing et al., 2020) |
|  |  |  | Defective lung formation, Peri-natal lethality | - | (Sah et al., 2020) |
|  | *Homo sapiens (RKO cell line)* | ΔDIP2c | Induction of epithelial-mesenchymal transition and enhanced cell motility |  | (Larsson et al., 2017) |
|  | *Homo sapiens* | DIP2a **^#^** | - | Developmental dyslexia | (Kong et al., 2016; Poelmans et al., 2009) |
|  |  |  | - | Autism | (Egger et al., 2014; Iossifov et al., 2012) |
|  |  | DIP2b **^#^** | - | Coronary artery disease | (Gong et al., 2018) |
|  |  | DIP2c **^#^** | - | Cancers | (Jiao et al., 2012; Rudin et al., 2012) |

#= SNP (single-nucleotide polymorphism), de novo frameshift/ nonsense variant, deletions
