# Supplementary material for: DIP2 is a unique regulator of diacylglycerol lipid homeostasis in eukaryotes
Source: eLife. 2022 Jun 29;11:e77665. doi: 10.7554/eLife.77665 (PMC9342972; doi:10.7554/eLife.77665)
Supplement: Supplementary file 2. [file elife-77665-supp2.docx]

**Supplementary Table 2:** List of yeast strains.

| **Strain ID** | **Genotype** | **Source** |
| --- | --- | --- |
| BY4741 | MATa his3∆1 leu2∆0 met15∆0 ura3∆0 | (Brachmann et al., 1998) |
| YSM57, YSM70  (∆ScDip2) | MATa; ura3Δ0; leu2Δ0; his3Δ1; met15Δ0; YOR093c::kanMX4 | This study |
| Y01869 (∆ScDip2) | MATa; ura3Δ0; leu2Δ0; his3Δ1; met15Δ0; YOR093c::kanMX4 | EUROSCARF |
| Y01907 (∆Ire1) | BY4741; MATa; ura3Δ0; leu2Δ0; his3Δ1; met15Δ0; YHR079c::kanMX4 | EUROSCARF |
| Y02501 (∆Dga1) | BY4741; MATa; ura3Δ0; leu2Δ0; his3Δ1; met15Δ0; YOR245c::kanMX4 | EUROSCARF |
| Y05383 (∆Lro1) | BY4741; MATa; ura3Δ0; leu2Δ0; his3Δ1; met15Δ0; YNR008w::kanMX4 | EUROSCARF |
| Y01608 (∆Dgk1) | BY4741; MATa; ura3Δ0; leu2Δ0; his3Δ1; met15Δ0; YOR311c::kanMX4 | EUROSCARF |
| YSM101 | MATa; ura3Δ0; leu2Δ0; his3Δ1; met15Δ0; YOR093c:: GFP(S65T)-KanMX6 | This study |
