# Supplementary material for: DIP2 is a unique regulator of diacylglycerol lipid homeostasis in eukaryotes
Source: eLife. 2022 Jun 29;11:e77665. doi: 10.7554/eLife.77665 (PMC9342972; doi:10.7554/eLife.77665)
Supplement: Supplementary file 3. [file elife-77665-supp3.docx]

**Supplementary Table 3:** List of Primers.

| **Name** | **Primer type** | **Primer sequences** |
| --- | --- | --- |
| CMR2-P1-FP | Forward | CAGCGTACGTTGCGTCTTA |
| CMR2-P2-RP | Reverse | CAATGACGATTGCAAAGAAGC |
| Kan-P3-FP | Forward | GTGACGACTGAATCCGGTG |
| CMR2-P4-RP | Reverse | CGCAGCTTCTCAGTGTTAC |
| CMR2-Del-5’-FP | Forward | GTGTTAGGGTGATTCAGTTCTGTGTAAAAGCGTGTGGCATTGAGTTACTCCAATGCGTACGCTGCAGGTCGAC |
| CMR2-Del-5’-RP | Reverse | CACAACTCAGTAGCATCCAATAGTCATGACAAATTTACTGTACTTGGATGTGTTAATCGATGAATTCGAGCTCG |
| CMR2-pGAL1- FP | Forward | GTCAAGGAGAAAAAACCCCATTAATTAAATGGATTTTTCTATTCCTCCTACC |
| CMR2-pGAL1-RP | Reverse | AGTACAGGTTTTCTCCGGACTCGAGAATATTGTCCTTTTCATAATCTGATAATAAATAAATGGAAATATTTTCAC |
| ScDMAP1-PGAL1-Gib-RP | Reverse | GAAGTACAGGTTTTCTCCGGACTCGAGTGCTGAGTCTCCACTGCTAGTATTTTCG |
| ScF2-pGAL1-Gib-FP | Forward | GTCAAGGAGAAAAAACCCCATTAATTAAATGGTTAAACCAAAACTTGCCCTACAATGC |
| ScF1F2-pGAL1-Gib-FP | Forward | GTCAAGGAGAAAAAACCCCATTAATTAAATGACGGATTCTTTACCGCTAATTTTACG |
| ScF1-pGAL1-Gib-RP | Reverse | GAAGTACAGGTTTTCTCCGGACTCGAGGAGATCATTGTTTAAAAACTTCTTCTCTACCGTG |
| PCMR2T-pPM90-FP | Forward | ACCTTGCATGCTCTCTACAATTAGCTTGTCTTTTC |
| PCMR2T-pPM90-RP | Reverse | AGCTCGGTACCAAGTTCGTTAAGGAAGAAACAAGAT |
| ScDip2-GFP-KanMX6-KI-FP | Forward | GGCATAAACTATGGTGAAAATATTTCCATTTATTTATTATCAGATTATGAAAAGGACAATATTGGTGGCAGTAAAGGAGAAGAACTTTTCACTGG |
| ScDip2-GFP-KanMX6-KI-RP | Reverse | CCACAACTCAGTAGCATCCAATAGTCATGACAAATTTACTGTACTTGGATGTGATCGATGAATTCGAGCTCGTTTAAACTGGATGG |
| ScDIP2-D523A-FP | Forward | CCCATGTTAACGTTATTGGCTTTTGGTGGTATCTTTATATCTATAAGAGATCA |
| ScDIP2-D523A-RP | Reverse | CTTATAGATATAAAGATACCACCAAAAGCCAATAACGTTAACATGGGAGAATA |
| ScDIP2-L687A-FP | Forward | CAAATACCTACTTTATGAGAACCAAGGCTATGGGGTTTGTTCATAACGGAAAGAT |
| ScDIP2-L687A-RP | Reverse | CCGTTATGAACAAACCCCATAGCCTTGGTTCTCATAAAGTAGGTATTTGCAGGAC |
| ScDIP2-N1357A-FP | Forward | GTTATGTCTATCAACATCACTTCGCTCCGCTTATATCATTAAGGTCGTATCTG |
| ScDIP2-N1357A-RP | Reverse | CGACCTTAATGATATAAGCGGAGCGAAGTGATGTTGATAGACATAACTTATTT |
| ScDIP2-D1482A-FP | Forward | TTAAGCTATTTGAGAACTGGTGCTCTGGGCTTTATCAAAAACGTAAGTTG |
| ScDIP2-D1482A-RP | Reverse | CGTTTTTGATAAAGCCCAGAGCACCAGTTCTCAAATAGCTTAAAGTGTTAT |
