# Supplementary material for: DIP2 is a unique regulator of diacylglycerol lipid homeostasis in eukaryotes
Source: eLife. 2022 Jun 29;11:e77665. doi: 10.7554/eLife.77665 (PMC9342972; doi:10.7554/eLife.77665)
Supplement: Supplementary file 4. [file elife-77665-supp4.docx]

**Supplementary Table 4:** List of plasmids.

| **Plasmid ID** | **Description** | **Reference** |
| --- | --- | --- |
| pYSM8 | pPPM90-CEN-URA3-YcpLac33 | Dr. Palani Murugan (CSIR-CCMB) |
| pYSM1 | pLE124 | Dr. Palani Murugan (CSIR-CCMB) |
| pYSM6 | pFA6a-GFP(S65T)-KanMX6 | Dr. Venkat Chalamcharla (CSIR-CCMB) |
| pYSM5 | pGAL1-MCS-TEV-GFP-8XHis-2XHA | This study |
| pYSM7 | pPPM90-PROMOTER-ScDIP2-TERMINATOR | This study |
| pYSM10 | pGAL1-ScDIP2-TEV-GFP-8XHis-2XHA | This study |
| ScDIp2 Domain truncated constructs | pGAL1-ScDBD1-TEV-GFP-8XHis-2XHA | This study |
|  | pGAL1-ScDBD1-TEV-GFP-8XHis-2XHA | This study |
|  | pGAL1-ScFLD1-TEV-GFP-8XHis-2XHA | This study |
|  | pGAL1-ScFLD2-TEV-GFP-8XHis-2XHA | This study |
|  | pGAL1-ScFLD1FLD2-TEV-GFP-8XHis-2XHA | This study |
|  | pGAL1-ScDBD1FLD1-TEV-GFP-8XHis-2XHA | This study |
| pPM47 (4xUPRE-RFP) | pPM47 (UPR-RFP CEN/ARS URA3) | (Merksamer et al., 2008) |
| pSM1960 | pRS426-SEC63-mRFP | (Metzger et al., 2008) |
